# Relationship Between Depression and Constipation: A Cross‐Sectional Health Report From the PERSIAN Guilan Cohort Study (PGCS)

**DOI:** 10.1002/hsr2.72804

**Published:** 2026-07-14

**Authors:** Maryam Ferdowsi, Niloofar Faraji, Saman Maroufizadeh, Fariborz Mansour‐Ghanaei, Farahnaz Joukar

**Affiliations:** ^1^ Gastrointestinal and Liver Diseases Research Center Guilan University of Medical Sciences Rasht Iran; ^2^ Department of Biostatistics, School of Health Guilan University of Medical Sciences Rasht Iran

**Keywords:** Cohort, constipation, depression, PERSIAN

## Abstract

**Background and Aims:**

The interaction between psychological factors and gastrointestinal function has been widely recognized, with particular attention given to how stress and anxiety can significantly influence digestive processes and bowel activity. This study evaluated the association between depression and constipation in the Prospective Epidemiological Research Studies in Iran (PERSIAN) Guilan Cohort Study (PGCS) population.

**Methods:**

This cross‐sectional study has been conducted on recorded data of 10520 participants aged 35–70 years. Demographic, lifestyle, and medical history data were collected through in‐person interviews and standardized questionnaires. Variables included age, sex, education, wealth index, tobacco and substance use, dietary habits, chronic disease history, and medication use. Depression was identified based on self‐reported physician diagnosis or current antidepressant use. Chronic constipation was defined as fewer than three bowel movements per week, assessed via questions on bowel habits, bloating, and laxative use, consistent with Mayo Clinic criteria. Data was analyzed using SPSS version 16.0 and level of significance was set at 0.05.

**Results:**

Among 10520 participants (mean age 51.52 ± 8.90 years; 53.5% female), the prevalence of depression and constipation was 5.22% and 4.45%, respectively. Depression was more common in females than males (7.33% *vs*. 2.78%, *p* < 0.001), while constipation was also more frequent in females (6.00% *vs*. 2.66%, *p* < 0.001) and increased with age (*p* < 0.001). Constipation was significantly more prevalent in those with depression (10.20% *vs*. 4.13%, *p* < 0.001), with a stronger association observed in males. Logistic regression showed that depression increased the odds of constipation (unadjusted OR = 2.64; adjusted OR = 1.92, 95% CI: 1.41–2.60), with a higher effect size in men across all models.

**Conclusion:**

These findings demonstrated that depression was significantly associated with higher odds of constipation, particularly among males, highlighting the need for integrated mental and gastrointestinal health assessments.

**Abbreviations**
CI: Confidence Interval
CRF: Corticotropin‐releasing factor
GERD: Gastroesophageal Reflux Disease
GI: Gastrointestinal
HPA: Hypothalamic‐pituitary‐adrenal
MI: Myocardial Infarction
MS: Multiple Sclerosis
OR: Odds Ratio
PERSIAN: Prospective Epidemiological Research Studies in Iran
PGCS: PERSIAN Guilan Cohort Study
SD: Standard Deviations

## 1 Introduction

Constipation is a prevalent gastrointestinal disorder among adults in the general population, with a global prevalence of approximately 15% [1]. Various risk factors for constipation have been identified, including demographic and socioeconomic factors, behavioral and lifestyle factors, and factors related to health status or underlying diseases [2, 3]. Among these factors, the role of psychological disorders, including depression, anxiety, and sleep disorders, has also been emphasized [4, 5], as the role of mental disorders such as depression and anxiety in bowel function is well recognized in scientific literature and is not unknown to researchers [4, 6, 7]. Depression is a common mental disorder worldwide, with a global lifetime prevalence of 14.6%, leading to significant impairments in individuals' professional, social, and personal lives [8, 9, 10]. Studies have shown that individuals with depression and anxiety are more likely to experience constipation and other gastrointestinal problems [6, 11]. This is because mental disorders can have a significant impact on bowel function, which is often referred to as the “gut‐brain axis” [12, 13].

However, regarding how mental disorders such as depression can affect bowel function, psychological factors, changes in serotonin levels, lifestyle, and certain medications can be mentioned. In particular, the imbalance in serotonin levels in individuals with depression and anxiety, which is increasingly important in regulating mood and bowel movements, is of great importance [13, 14, 15]. Investigating the relationship between constipation and depression is important from two perspectives. On the one hand, the prevalence of these two conditions is reported in a large population of the Iranian from an epidemiological point of view, and on the other hand, considering the two‐way path of the gut‐brain axis [12], constipation and depression can be investigated for their bidirectional association and potential to exacerbate one another [13, 16].

Given the differences in the results of studies related to the investigation of the relationship and factors affecting the development of depression and the occurrence of constipation in different regions, and the physiological and socioeconomic differences of the inhabitants of these regions, there is a need for new studies in this field. In this study, we aimed to investigate the relationship between depression and factors affecting the development of constipation in the Prospective Epidemiological Research Studies in Iran (PERSIAN) Guilan Cohort Study (PGCS) population because of its large sample size and large number of variables provide valuable evidence.

## 2 Methods

### 2.1 Participants and Study Design

This analytical cross‐sectional study utilized data from the PGCS, a large population‐based cohort study comprising 10520 male and female participants aged 35–70 years (Sowme'eh Sara, Guilan, Iran). At the baseline visit, the participants completed questionnaires eliciting clinical data, lifestyle characteristics, and demographic information. Data collection also included a physical examination and a structured questionnaire. Comprehensive details regarding the PGCS profile and the PERSIAN protocol have been published previously [17, 18].

### 2.2 Data Collection and Variable Measurement

Demographic data collected included age, sex, marital status, education level, wealth score index (WSI), body mass index (BMI), physical activity, occupation, habitat, history of cigarette and hookah use, opium use, alcohol consumption, tea and coffee intake, and vegetable and fruit consumption (per week). Anthropometric measurements including height (cm) and weight (kg) are presented in Table 1. Medical history data, encompassing chronic diseases such as hypertension, diabetes, cancer, cardiac ischemia, myocardial infarction (MI), stroke, renal failure, chronic lung disease, thyroiditis, rheumatic disease, epilepsy, chronic headache, multiple sclerosis (MS), and medication use, were collected via in‐person interviews and pre‐existing questionnaires.

**Table 1:** Demographic and clinical characteristics of the participants in the Persian Guilan Cohort Study (*n* = 10520).

Variable	Total	Non‐Depression group	Depression group	*p*
	Mean (SD) or *n* (%)	Mean (SD) or *n* (%)	Mean (SD) or *n* (%)	
Age (years)	51.52 ± 8.90	51.49 ± 8.90	52.08 ± 8.92	0.130
35–45	3139 (29.8)	2982 (29.9)	157 (28.6)	
45–55	3854 (36.6)	3664 (36.7)	190 (34.6)	
> 55	3527 (33.5)	3325 (33.3)	202 (36.8)	
Sex				< 0.001
Male	4887 (46.5)	4751 (47.6)	136 (24.8)	
Female	5633 (53.5)	5220 (52.4)	413 (75.2)	
Marital status				< 0.001
Single	305 (2.9)	289 (2.9)	16 (2.9)	
Married	9527 (90.6)	9054 (90.8)	473 (86.2)	
Widow	566 (5.4)	519 (5.2)	47 (8.6)	
Divorced	122 (1.1)	109 (1.1)	13 (2.4)	
Education level	6.63 ± 4.52	6.63 ± 4.52	6.56 ± 4.54	0.728
Illiterate	1738 (16.5)	1645 (16.5)	93 (16.9)	
1–5	3312 (31.5)	3135 (31.4)	177 (32.2)	
6–12	4832 (45.9)	4584 (46.0)	248 (45.2)	
University	638 (6.1)	607 (6.1)	31 (5.6)	
Employment				< 0.001
Unemployed	4781 (45.4)	4419 (44.3)	362 (65.9)	
Employed	5739 (54.6)	5552 (55.7)	187 (34.1)	
Habitat				0.878
Urban	4613 (43.8)	4374 (43.9)	239 (43.5)	
Rural	5907 (56.2)	5597 (56.1)	310 (56.5)	
Wealth score index (z score)	0 ± 1	0.01 ± 1.01	−0.06 ± 0.98	0.154
Physical activity	41.26 ± 8.88	41.43 ± 8.92	38.19 ± 7.55	< 0.001
BMI (kg/m2)	28.14 ± 5.09	28.09 ± 5.07	29.10 ± 5.23	< 0.001
Underweight	141 (1.3)	137 (1.4)	4 (0.7)	
Normal	2746 (26.1)	2632 (26.4)	114 (20.8)	
Overweight	4198 (39.9)	3978 (39.9)	219 (39.9)	
Obese	3435 (32.7)	3224 (32.3)	212 (38.6)	
Smoking	2584 (24.6)	2494 (25.0)	90 (16.4)	< 0.001
Hookah consumption	1515 (14.4)	1457 (14.6)	58 (10.6)	0.009
Opium consumption	726 (6.9)	701 (7.0)	25 (4.6)	0.026
Alcohol consumption	1395 (13.3)	1327 (13.3)	68 (12.4)	0.535
Number of chronic diseases				< 0.001
0	3196 (30.4)	3099 (31.1)	97 (17.7)	
1	3470 (33.0)	3301 (33.1)	169 (30.8)	
2	2270 (21.6)	2139 (21.5)	131 (23.9)	
> 3	1584 (15.1)	1432 (14.4)	152 (27.7)	
Tea	7663 (72.8)	7270 (72.9)	393 (71.6)	0.496
Coffee	4578 (43.6)	4348 (43.6)	239 (43.5)	0.973
Vegetables (per week)				0.046
< 3	949 (9.0)	895 (9.0)	54 (9.8)	
3–5	2935 (27.9)	2761 (27.7)	174 (31.7)	
> 5	6636 (63.1)	6315 (63.3)	321 (58.5)	
Fruits (per week)				0.004
< 2	1577 (15.0)	1483 (14.9)	94 (17.1)	
2–4	3698 (35.2)	3481 (34.9)	217 (39.5)	
> 4	5245 (49.9)	5007 (50.2)	238 (43.4)	
NSAIDs	1505 (14.3)	1394 (14.0)	111 (20.2)	< 0.001
Anticholinergics drugs	16 (0.2)	13 (0.1)	3 (0.5)	0.048

Abbreviations: BMI, Body Mass Index; NSAIDs, Non‐steroidal anti‐inflammatory drugs; SD, Standard Deviation.

### 2.3 Depression Assessment

Depression status was determined through face‐to‐face interviews with the participants. The diagnostic criterion for depression was based on the individual's self‐report within their clinical history, including information regarding current and past medication use, as well as prior or current diagnosis and its confirmation by a psychiatrist. In essence, depression was assessed using participants' self‐reported data and was operationalized as a positive response to any of the following criteria [1]: a prior physician diagnosis of depression, or [2] current use of antidepressant medications.

### 2.4 Constipation Assessment

In this study, chronic constipation was defined as fewer than three bowel movements per week, consistent with the Mayo Clinic's diagnostic criteria and questionnaire [19]. Constipation was assessed via questions regarding current and past health conditions, including bowel movement frequency, laxative use, history of bloating and abdominal distension (particularly postprandial), and usual bowel habits. Participants who self‐reported constipation and used regular laxatives were classified as having constipation.

### 2.5 Statistical Analysis

Variables were presented as mean ± standard deviation (SD) and number (percentage). Differences in continuous and categorical variables between participants with and without depression were tested by independent t‐test and chi‐square test (or Cochran–Armitage test for trend), respectively. The difference between the prevalence of the constipation between participants with and without depression was examined using Chi‐square test. The relationship between depression and constipation was examined using logistic regression analysis. Odds ratio (OR) and 95% confidence interval (CI) were calculated. OR was also adjusted for some demographic and clinical characteristics. In total, three models were analyzed. Model 1 was unadjusted and included only depression. Model 2 was adjusted for age, sex, marital status, years of education, occupation, place of residency, WSI, BMI, physical activity, smoking, hookah smoking, drug consumption, alcohol consumption, and number of chronic diseases. Model 3 was Model 2 plus adjustment for tea and coffee consumption, number of vegetables and fruit consumption per week, NSAIDs and anticholinergics drugs. All analyses were also performed by sex. Data analysis was carried out using SPSS for Windows, version 16.0 (SPSS Inc., Chicago, IL, USA), and level of significance was set at 0.05. Missing data across the analyzed variables were negligible and handled using complete‐case analysis to ensure methodological transparency.

## 3 Results

### 3.1 Characteristics of the Participants

Demographic and clinical characteristics of the participants are presented in Table 1. The mean age of the participants was 51.52 (SD = 8.90) years. Of the participants, 53.5% were female, 90.6% were married, 6.1% had university education, 45.4% were unemployed, 56.2% were resident in rural area, 32.7% had obese‐BMI, 24.6% were smokers, and 13.3% consumed alcohol. Comparisons of characteristics between subjects with and without depression were presented in Table 1. Compared to participants without depression, subjects with depression were predominantly female, more likely to be widowed or divorced, and more frequently unemployed. Furthermore, they reported lower rates of smoking, hookah use, and opium consumption. They also exhibited higher BMIs, lower physical activity levels, and reduced consumption of fruits and vegetables, and reported more use of NSAIDs and anticholinergics drugs.

### 3.2 Prevalence of Depression and Constipation

The prevalence of depression was 5.22% in this study. It was higher in females than in males (7.33% vs 2.78%, *p* < 0.001), and there was no relationship between age and depression (*p* = 0.247). The prevalence of constipation was 4.45% and was more common in females than in males (6.00% vs 2.66%, *p* < 0.001). The prevalence of constipation increased with age: the highest was 5.95% in those aged 55 or more years (*p* < 0.001) (Table 2).

**Table 2:** Prevalence of depression and constipation among the participants.

	Depression		Constipation	
	*n*/*N*	%	*n*/*N*	%
Total	549/10520	5.22	486/10520	4.45
Age				
35–44	157/3139	5.00	115/3139	3.66
45–54	190/3854	4.93	143/3854	3.71
≥ 55	202/3527	5.73	210/3527	5.95
*p* a	0.247		< 0.001	
*p* for trend^b^	0.172		< 0.001	
Sex				
Male	136/4887	2.78	130/4887	2.66
Female	413/5633	7.33	338/5633	6.00
*p* a	< 0.001		< 0.001	

^a^ Chi–Square test.

^b^ Cochran–Armitage test for trend.

### 3.3 Relationship Between Depression and Constipation

#### 3.3.1 Chi‐square test

**Figure 1:**
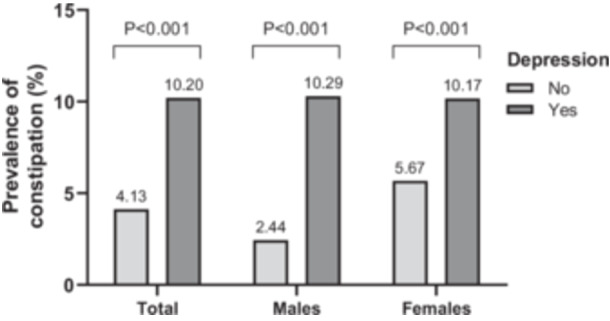
Prevalence of constipation based on the depression among the participants in the Persian Guilan Cohort Study. *p* values are based on the Chi‐square test.

The prevalence of constipation was significantly higher among participants with depression than those without (10.20% vs 4.13%, *p* < 0.001). This difference was also significant in subgroup analysis for males (10.29% vs 2.44%, *p* < 0.001) and females (10.17% vs 5.67%, *p* < 0.001), although the difference was higher in males (Table 3 and Figure 1).

**Table 3:** Relationship between depression and constipation among the participants in the Persian Guilan Cohort Study.

	Chi‐Square test			Logistic regression analysis					
	Prevalence of constipation			Model 1 (unadjusted)		Model 2		Model 3	
	*n*/*N*	%	*p*	OR (95% CI)	*p*	OR (95% CI)	*p*	OR (95% CI)	*p*
Total									
Depression			< 0.001						
No	412/9971	4.13		1		1		1	
Yes	56/549	10.20		2.64 (1.97–3.53)	< 0.001	1.95 (1.44–2.64)	< 0.001	1.92 (1.41–2.60)	< 0.001
Males									
Depression			< 0.001						
No	116/4751	2.44		1		1		1	
Yes	14/136	10.29		4.59 (2.56–8.21)	< 0.001	3.52 (1.90–6.51)	< 0.001	3.47 (1.86–6.44)	< 0.001
Females									
Depression			< 0.001						
No	296/5220	5.67		1		1		1	
Yes	42/413	10.17		1.88 (1.34–2.65)	< 0.001	1.67 (1.18–2.36)	0.004	1.65 (1.16–2.35)	0.005

*Note:* Model 1: Unadjusted model. Model 2: Adjusted for age and sex, marital status, education, employment, place of residency, wealth score index, physical activity, smoking, hookah smoking, opium consumption, and alcohol consumption, and number of chronic diseases. Model 3: Adjusted for variables in Model 2 plus tea consumption, coffee consumption, number of vegetables and fruit consumption, NSAIDs, and anticholinergics drugs.

Abbreviations: CI, confidence interval; OR, odds ratio.

### 3.4 Logistic Regression Analysis

In unadjusted model, presence of depression increased the odds of constipation by 2.64‐fold (OR = 2.64, 95% CI: 1.97–3.53, *p* < 0.001) (Model 1). After adjustment for some demographic and clinical variables in Model 2, OR was decreased to 1.95 (95% CI: 1.44–2.64, *p* < 0.001) (Model 2). After additional adjustment for other variables in Model 3, this association remained significant (OR = 1.92, 95% CI: 1.41–2.60, *p* < 0.001) (Table 3).

Logistic regression analyses were also performed to examine the association between depression and constipation by gender. In unadjusted model (Model 1), the relationship between depression and constipation in males (OR = 4.59; 95% CI: 2.56–8.21) was stronger than that in females (OR = 1.88; 95% CI: 1.34–2.65). Similar results were also obtained after adjustment for variables in Model 2 and Model 3 (Table 3).

## 4 Discussion

This study presented the first report from northern Iran on the prevalence of depression and constipation within the large population of the PGCS. Both conditions were relatively common in the study population. Notably, the prevalence of constipation was significantly higher in individuals with depression compared to those without, across both genders. Specifically, depression increased the odds of experiencing constipation by 2.64 times. A critical finding was that the depression‐constipation association was stronger in men than in women, highlighting gender as a key factor in the diagnosis and progression of psychiatric disorders, including depression.

The overall constipation prevalence was 4.45% (2.6% in men, 6% in women), increasing with age and reaching 5.95% in individuals aged ≥ 55 years (*p* < 0.001). This underscores the role of aging in reduced gastrointestinal motility and heightened constipation risk [20], The age‐related increase in constipation prevalence aligns with findings from numerous previous studies [21, 22]. Older age, physical inactivity, opium use, anxiety, depression, insomnia, back pain or arthralgia, and gastroesophageal reflux disease (GERD) have been reported as major risk factors for constipation [23].

Reported constipation rates in this study were lower than in previous Iranian and Western studies. A study by Adibi et al. reported 23.9% prevalence in Iran [7], while Moezi et al. found 8.1% [23], with higher rates in women. A systematic review of Iranian studies estimated chronic constipation prevalence between 2.4% and 11.2% [24]. Comparatively, population‐based studies in North America reported an average prevalence of 14.5% [25], while a meta‐analysis study noted 16% in Northern/Southern Europe, 14% in the Middle East, and 11% in Southeast Asia [26]. These disparities may stem from differences in lifestyle, high‐fiber diets, and diagnostic tools for constipation. Notably, the highest prevalence rates are often observed when self‐report questionnaires are used [21, 27].

In the current study, the prevalence of depression was 5.22%, with no significant age‐based differences. Geographically, the highest and lowest rates were in central Iran (41.7%) and southern Iran (21.9%), respectively, possibly due to uneven distribution of psychiatrists, treatment centers, and mental health resources [28]. Global estimates vary due to differing measurement tools, sample sizes, and different methodological approaches to assessing depression [29]. Regarding ethnicity, cultural traditions in certain ethnic groups may limit individuals' activity levels, potentially creating conditions conducive to depression. Socioeconomic status is another key factor contributing to variations in the prevalence of mental disorders across regions, with studies indicating higher rates in less developed or economically disadvantaged areas [30, 31].

Moreover, we found that women exhibited higher rates of both depression and constipation, aligning with studies suggesting greater susceptibility to mood disorders and functional gastrointestinal issues [32]. Evidence showed that women with IBS more frequently report constipation, whereas men report diarrhea [32]. Overall, the higher prevalence of constipation in women may be attributed to hormonal fluctuations, differences in dietary habits, or specific gender‐related physiological factors [33]. Globally, women are 1.7 times more likely to experience depression than men during their lifetime [34], a trend attributed to sex‐specific physiological and biological differences, and less dependent on race, culture, diet, education, and many other potential confounding socioeconomic factors [35]. This may be related to this fact that females are typically more sensitive than males and more affected by surrounding events [36].

Evidence have shown significant sex differences in the expression of genes related to depression in men and women, which could explain the variations in prevalence and pathophysiology of the disease between the sexes [37]. Furthermore, hormonal factors are among the most influential factors in the development of mental disorders in women. Given that the peak onset of depressive disorders in women coincides with their reproductive years, hormonal risk factors may play a significant role in depression [38].

Although a higher prevalence of depression has been reported among women both in this study and globally, the increased odds of constipation among men with depression observed in the present study further highlight the role of sex in gastrointestinal disorders. Notably, logistic regression analysis revealed a stronger association between depression and constipation in men (OR = 4.59; 95% CI: 2.56–8.21) compared to women (OR = 1.88; 95% CI: 1.34–2.65), supporting previous findings in Iranian adults aged 18 to 55 years [7].

Although no studies to date have conclusively confirmed sex differences in the association between constipation and depression across diverse populations, this possibility cannot be ruled out. This observation may be partly explained by the complexity and diversity of the gut microbiota, which is influenced by sex. Emerging evidence suggests that variations in gut microbiota may directly or indirectly affect steroid hormone regulation and gene expression. Increasing attention has been directed toward the role of the gut microbiota in maintaining mental health and its involvement in the pathogenesis of various diseases. Overall, current evidence indicates that the male brain may be particularly vulnerable to early‐life microbial influences. The gut microbiota has also been shown to affect the composition of sex hormones and bile acid levels [33]. Therefore, sex appears to be an important factor not only in the diagnosis and development of certain psychiatric disorders, including depression, but also in shaping gut microbiota profiles, which may in turn contribute to these conditions.

After adjusting for sociodemographic, lifestyle, and medical factors, the depression‐constipation association persisted, suggesting depression is an independent risk factor for constipation. These findings are consistent with previous studies that have shown a significant association between depression and constipation [39, 40]. This aligns with the gut‐brain axis hypothesis. Clinically, depression is associated with dysregulation of the hypothalamic‐pituitary‐adrenal (HPA) axis, and activation of the HPA axis releases stress factors such as corticotropin‐releasing factor (CRF), which affects gastrointestinal function, consequently leading to constipation [41, 42]. Sustained activation of the stress pathways mentioned above may lead to dysfunction in the brain‐gut axis, predisposing depressed patients to symptoms such as chronic diarrhea or chronic constipation [6].

Furthermore, it should be noted that psychiatric disorders, such as depression, may also be associated with constipation due to the direct impact of the illness itself or other related factors. Patients with depression and other psychiatric disorders commonly have unhealthy lifestyles, such as poor diet, inadequate fluid intake, and lack of physical activity, which may also contribute to the development of constipation [43].

The robust depression‐constipation link warrants further investigation into underlying mechanisms, particularly gut‐brain axis interactions and sex‐specific factors. Longitudinal studies could clarify temporal relationships. Despite variations, this association remains consistent across studies, emphasizing the need for gender‐sensitive approaches in mental and gastrointestinal health research. So, longitudinal studies could help determine whether depression precedes constipation or if the relationship is bidirectional.

This study has several notable strengths and limitations. A primary strength is the large, population‐based sample size of the PGCS, which significantly strengthens the statistical precision of our estimates. However, the results must be interpreted within the context of certain limitations. First, due to the cross‐sectional nature of the data, we cannot establish temporal or causal relationships between depression and constipation. Second, depression was defined based on self‐reported physician diagnosis and current medication use rather than a validated diagnostic clinical scale, which introduces the possibility of misclassification bias. Finally, while our regression models adjusted for a wide array of demographic and clinical variables, the potential for residual confounding from unmeasured factors—such as specific dietary fiber intake quantities or gut microbiome variations remains. Therefore, longitudinal studies could help determine whether depression precedes constipation or if the relationship is bidirectional.

## 5 Conclusion

This study strengthens the understanding of the intricate relationship between mental and gastrointestinal (GI) health. Our findings demonstrate that depression remains a significant predictor of constipation even after accounting for potential confounding variables. Moreover, the observed gender‐specific effects underscore the necessity for tailored approaches in future research and clinical practice, suggesting that the underlying mechanisms linking depression and constipation may differ between sexes. From a clinical perspective, these results highlight the importance of proactively screening individuals with depression for GI symptoms, particularly constipation, and conversely, assessing individuals presenting with constipation for potential depressive symptoms. Early identification and appropriate management of these co‐occurring conditions have the potential to improve patient outcomes and overall quality of life.

## Author Contributions

**Maryam Ferdowsi:** investigation, writing – original draft, methodology, validation, writing – review and editing, data curation. **Niloofar Faraji:** writing – original draft, investigation, writing – review andediting, data curation. **Saman Maroufizadeh:** writing – review and editing, methodology, software, data curation, formal analysis. **Fariborz Mansour‐Ghanaei:** conceptualization, data curation, supervision, writing – review and editing, validation, methodology, investigation. **Farahnaz Joukar:** conceptualization, supervision, data curation, validation, methodology, software, formal analysis, investigation, writing – original draft, writing – review and editing.

## Funding

The authors have nothing to report.

## Ethics Statement

This research was approved by the Ethics Committee of Guilan University of Medical Sciences, Rasht, Iran (Ethics Code: IR.GUMS.REC.1403.442), and all participants provided written informed consent by signing a form. In cases where the research participants are unable to sign a form, because of a language barrier, then oral or verbal consent have been obtained and documented in the presence of a witness. In the case of adults with cognitive decline, a parent and/or legal guardian/family or an appropriate representative gave informed consent to participate on their behalf.

## Conflicts of Interest

The authors declare no conflicts of interest.

## Transparency Statement

The corresponding authors affirm that this manuscript is an honest, accurate, and transparent account of the study being reported; that no important aspects of the study have been omitted; and that any discrepancies from the study as planned (and, if relevant, registered) have been explained.

## Data Availability

The datasets used and/or analyzed in the current study are available from the corresponding author on reasonable request.
